# Hepatobiliary Cancers in Saudi Arabia From 2000 to 2025

**DOI:** 10.7759/cureus.81994

**Published:** 2025-04-10

**Authors:** Khalil I Alshammari, Ibrahim Ginawi, Hisham Sherfi, Hussain G Ahmed

**Affiliations:** 1 Internal Medicine, Imam Mohammad Ibn Saud Islamic University, Riyadh, SAU; 2 Public Health, Ministry of Health, Ḥaʼil, SAU; 3 Medicine, University of Ha'il, Ha'il, SAU; 4 Pathology, Prof Medical Research Consultancy Center, El-Obeid, SDN; 5 Histopathology and Cytology, Faculty of Medical Laboratory Sciences, University of Khartoum, Khartoum, SDN

**Keywords:** cholangiocarcinoma, hepatobilary cancers, hepatocellular carcinoma, liver cancer, saudi arabia

## Abstract

Hepatobiliary cancers present a significant challenge to global health. Saudi Arabia and adjacent Gulf nations experience considerable impacts from these cancers. Numerous risk factors have contributed to the increasing prevalence of these cancers. The primary cases are linked to several factors, including hepatitis viral infection, smoking, alcohol consumption, diabetes mellitus, obesity or being overweight, liver cirrhosis, nonalcoholic fatty liver disease, hemochromatosis, aflatoxins, anabolic steroids, and genetic predisposition. Data regarding hepatobiliary cancers is scarcely obtained from Saudi Arabia. This research aims to clarify the epidemiology and risk factors linked to hepatobiliary cancers in Saudi Arabia. Our investigation revealed a lack of studies that collectively examine hepatocellular cancers in Saudi Arabia, highlighting a distinctive element of the current review. To determine the incidence, prevalence, risk factors, and other epidemiological metrics of hepatobiliary cancer in Saudi Arabia, a search was conducted using Medline/PubMed, Scopus, Web of Knowledge, Google Scholar, and relevant public databases that fulfilled the inclusion criteria. An electronic search was conducted using various keywords related to hepatobiliary cancer in Saudi Arabia. In summary, hepatobiliary cancers exhibit significant prevalence in Saudi Arabia, especially liver cancer. Commonly recognized risk factors for type 2 diabetes mellitus encompass tobacco and alcohol consumption, obesity or overweight status, and viral hepatitis.

## Introduction and background

Hepatobiliary malignancies encompass a wide range of invasive carcinomas arising from the bile ducts (both intrahepatic and extrahepatic cholangiocarcinoma (CCA)), the liver (hepatocellular carcinoma (HCC)), and the gallbladder [1].

HCC is a significant global health concern, ranking as the fourth leading cause of cancer-related mortality worldwide. HCC represents the predominant form of primary liver cancer (LC). Long-term viral infections such as hepatitis B virus (HBV) and hepatitis C virus (HCV), nonalcoholic steatohepatitis (NASH), excessive alcohol consumption, and other conditions can lead to liver swelling and scarring [2].

Biliary tract cancer (BTC) includes a variety of aggressive malignancies that arise from the epithelium of the biliary tract. A significant number of patients receive diagnoses of locally advanced or metastatic disease [3]. CCA is a malignancy of the bile duct characterized by late-stage diagnosis, unfavorable prognosis, and resistance to pharmacological treatment. CCAs are primarily defined by their anatomical location and exhibit molecular subclasses that demonstrate both inter- and intratumoral variability. Besides tumor cells, CCA features a complex and dynamic tumor microenvironment characterized by intricate interactions between tumor and stromal cells. Cancer-associated fibroblasts (CAFs) are present in the stroma of a CCA tumor. These cells modify the extracellular matrix (ECM), influence the immune system, generate new blood vessels, and facilitate cancer dissemination. Recent studies indicate that CAFs consist of transcriptionally and functionally diverse subgroups that both promote and inhibit tumor progression [4].

Recent data indicate a rise in cancer incidence in Saudi Arabia. Research initiatives throughout the cancer continuum are crucial for improving control strategies. As cancer research advances in the country, identifying existing challenges and implementing targeted interventions to address them is crucial. Improving health research is a primary goal of Saudi Vision 2030. Addressing the existing challenges in cancer research is crucial for ensuring that research outcomes effectively guide policies concerning cancer control and care delivery [5]. However, the terminology related to hepatobiliary cancers is rarely utilized in Saudi Arabia. Health practitioners and policymakers often consider these conditions as separate entities, including LC, gallbladder cancer, and bile duct cancer. This leads to the formulation of specific guidelines for each entity.

Saudi Arabia has limited epidemiological data on hepatobiliary cancers, with the notable exception of HCC. The country demonstrates a high incidence of HCC, often diagnosed at advanced stages, leading to unfavorable outcomes. A substantial number of HCC cases were linked to viral hepatitis, with a considerable fraction also exhibiting liver cirrhosis [6].

The shift in Saudi lifestyle toward Western dietary practices in recent decades has resulted in a heightened overall cancer burden, especially concerning gastrointestinal cancers. This review aims to provide epidemiological data on hepatobiliary cancers in Saudi Arabia.

## Review

Data and search methods

The literature on hepatobiliary cancers in Saudi Arabia was gathered through an electronic search across multiple databases, including Medline/PubMed, Cochrane Library, Scopus, Web of Knowledge, Google Scholar, and public databases such as GLOBOCAN 2022 and the International Agency for Research on Cancer (IARC), all of which fulfilled the inclusion criteria. Data were also obtained from the Saudi Cancer Registry (SCR). Specific keywords related to hepatobiliary cancers were used in the search, including hepatobiliary.

Inclusion criteria

Only publications from Saudi Arabia between 2000 and 2025 that focused on the epidemiology of hepatobiliary cancers and their associated risk factors were included, as well as data from the Saudi National Cancer Registry.

Exclusion criteria

Publications in languages other than English, as well as those focusing on hepatobiliary cancer survivors and qualitative studies, were excluded, as were papers relating to laboratory research using animal experiments.

LC (HCC)

LC, the sixth most prevalent cancer globally and the second leading cause of cancer-related mortality, constitutes a significant public health concern. The diagnosis of advanced illness correlates with mortality rates [7]. HCC is the leading cause of cancer-related mortality and morbidity among people with chronic liver disease and cirrhosis globally. The increasing global prevalence of nonalcoholic fatty liver disease (NAFLD) and the escalating alcohol intake in numerous nations are shifting the primary etiology of HCC from viral to nonviral origins. Although recent progress in HCC treatment is promising, decreasing HCC mortality necessitates early detection, ongoing surveillance, and equitable access to HCC therapeutics [8].

HCC, the predominant histological variant of LC, accounts for the majority of diagnoses and fatalities. HBV and HCV are the principal global risk factors for HCC; however, their prevalence may diminish in the forthcoming years. The impact of neonatal HBV vaccination, already seen in young adults across several nations, will increase as the vaccinated cohorts mature. Efficient therapies for chronic HBV and HCV infections ought to diminish viral-associated HCC. Regrettably, metabolic risk factors for HCC, including metabolic syndrome, obesity, type II diabetes, and NAFLD, are increasing and may emerge as the predominant cause of HCC globally. Aflatoxin contamination of food crops and excessive alcohol consumption persist as risk factors in certain nations. Although early diagnosis and improved therapy are crucial for HCC, primary prevention strategies aimed at mitigating obesity, diabetes, and mycotoxin proliferation are as significant [9].

Cirrhosis constitutes the principal risk factor for HCC. However, the transition in the etiology of HCC from viral liver diseases to nonviral origins, such as alcoholic steatosis and metabolic dysfunction, significantly influences the strategies for prevention, surveillance, and management of HCC. Antiviral agents for HBV and HCV, together with HBV vaccination, can mitigate the risk of virus-induced HCCs; still, chemoprevention for nonviral liver diseases remains essential. Aspirin, statins, metformin, and coffee may reduce the incidence of HCC; however, no clear correlation has been established. Biannual monitoring enhances the early diagnosis of HCC; however, existing modalities, such as abdominal ultrasonography, exhibit limited efficacy in identifying early-stage HCC, particularly in individuals with obesity and/or nonviral liver diseases. Blood-based or imaging-based surveillance methods, despite their promise, necessitate additional validation prior to implementation in clinical practice. Concurrently, we ought to optimize current monitoring capabilities, considering their global underutilization. The therapy of HCC has advanced, resulting in increased surgical eligibility for patients, enhanced local patient selection, and a broader array of systemic therapeutic options, including immune checkpoint inhibitors [10].

Biliary tract

The biliary tract consists of the gallbladder and the intrahepatic and extrahepatic biliary trees. The principal duodenal papilla delivers bile to the second portion of the duodenum. Cholangiocytes make up the epithelium of the biliary tract. Malignancies arising from the cholangiocyte-rich bile duct epithelium result in BTC. These cancers are classified based on their anatomical location: intrahepatic CCA and extrahepatic CCA. Klatskin tumors, or perihilar malignancies, are a form of extrahepatic CCA that originate from the bile duct epithelium at the junction of the right and left hepatic ducts, along with the cystic duct leading to the common bile duct. Additionally, distant CCA affects the gallbladder, the ampulla of Vater, and the pancreatic biliary channels.

Both intrahepatic and extrahepatic CCA arise from the bile duct epithelium, though their anatomical locations influence their progression and clinical outcomes. CCA is a rare and highly malignant disease with a poor prognosis, as it is usually diagnosed at an advanced and unresectable stage. The tumor’s delayed presentation often involves blood vessels and adjacent lymph nodes, making curative surgical resection difficult. Although uncommon, CCA is the second most prevalent primary LC (PLC), following HCC [11].

Hepatobiliary cancers in Saudi Arabia

Primary hepatic carcinoma (PHC) is the fourth most common cancer among men in Saudi Arabia. According to the SCR, the incidence of PHC increased in Saudi Arabia from 2001 to 2014, from 323 cases in 2001 to 376 cases in 2015. From 1975 to 2014, King Faisal Specialist Hospital and Research Center reported 2,779 new cases of PHC. The incidence of PHC increased from 60 cases in 2004 to 80 in 2014. HCC is the most common type of LC, accounting for 79.3% of occurrences, followed by CCA (11%) and hepatoblastoma (4.7%). Males have a considerably higher incidence compared to females, with a 2:1 ratio (p < 0.01). The highest prevalence occurred between the sixth and seventh decades of life. A considerable majority of patients (44.6%) were diagnosed at the localized stage, and 28.2% had a history of hepatitis (p < 0.001) [12].

Intermediate- and advanced-stage HCC pose a significant healthcare burden in Saudi Arabia, yet little is known about its unmet needs and treatment options. HCC accounted for the majority of LC cases due to increased chronic viral infections and lifestyle risk factors. Most Saudi doctors utilize the Barcelona Clinic Liver Cancer Criteria to detect and stage HCC. Most Saudi HCC patients are diagnosed in the intermediate or advanced stages, which results in a poor prognosis and restricted therapeutic options. To address HCC in Saudi Arabia, evidence-based surveillance, multidisciplinary diagnosis, and more treatment options are required [13]. Epidemiological studies of liver and intrahepatic CCA in the Middle East and North Africa region show that LC, particularly HCC, is on the rise. This increase is due to a combination of viral hepatitis, underlying metabolic problems, and environmental factors. The epidemiology of LC varies significantly among locations. For example, Egypt has long been affected by HCV, whereas the Gulf states are seeing an increase in cases related to metabolic problems. Forecasts indicate a sustained increase in LC cases as a result of the ongoing obesity and diabetes crises [14].

According to the Global Cancer Observatory, the overall incidence of hepatobiliary malignancies in 2022 is 5.9%, with LC accounting for 4.5%, gallbladder cancer at 1.2%, and hepatic bile duct cancer at 0.2%. The researchers calculated a cumulative risk of 0.66, with 0.57 attributed to LC and 0.09 to gallbladder cancer. Over a five-year period, the cumulative frequency was 5.77 per 100,000 people, with 4.9 ascribed to liver disease and 0.87 to gallbladder problems. The mortality rate associated with hepatobiliary cancers is 10.3%, with 9.2% for LC and 1.1% for gallbladder cancer. Saudi men are more likely to get hepatobiliary malignancies. LC is the fourth most common cancer in Saudi Arabia [15]. Despite advances in preventive interventions, the incidence of PHC has increased over the last decade, with significant regional variation. Incidence rates vary significantly across Saudi Arabia. The diversity of lifestyles in these regions helps to explain this difference. This growing trend has multiple causes, including chronic infection with HBV and/or HCV, excessive alcohol consumption, obesity, diabetes, and tobacco use. The development of healthcare delivery in the Kingdom may have improved early identification and diagnosis, adding to the exponential rise. More studies are needed to better understand the rising patterns at the molecular and genetic levels [12].

In most countries, men are two to three times more likely than women to develop and die from LC. LC is the second leading cause of male death worldwide [16]. Significant global inequalities in HCC incidence and mortality are described. Thailand, Vietnam, and Cambodia each reported 22-24 cases and deaths per 100,000 persons in 2020. HCC is most prevalent in China, followed by Japan, Thailand, and Vietnam. Patients beyond the age of 80 are more likely to be diagnosed later, receive fewer treatments, and have shorter survival rates than those under the age of 60. Men were at a higher risk of developing LC than women, with an HR of 3.9 (95% CI: 3.6-4.2) for HCC, 1.2 (95% CI: 1.1-1.3) for CCA, and 1.7 (95% CI: 1.5-2.0) for other specified or unspecified LC. Asians and Black Africans have a higher incidence of HCC than white British. Poorer individuals were more likely to receive emergency diagnoses. Overall, survival was low. HCC patients showed a better 10-year survival rate (14.5%, 13.1-16.0%) compared to CCA (4.4%, 3.4-5.6%) and other liver tumors (12.5%, 10.1-15.2%). Survival rates for 62.7% of LC patients with missing or unclear stages ranged from Stage III to IV [17].

GLOBOCAN 2020 predicts a 55% and 56% increase in PLC cases and fatalities between 2020 and 2040. The gains were roughly twice as great in low HDI countries, indicating an aging population. GLOBOCAN 2020 provides one of the most comprehensive global LC estimates, but it will be interesting to see if additional data sources support the projected upward trend. The IARC predicted LC incidence and mortality by assuming national rates would remain constant from 2020 to 2040 (hereafter referred to as the IARC population projection), but with only one year of data available, they were unable to account for key trends [18-21].

A study gathered PLC cases and fatalities from the GLOBOCAN 2020 database, which covers 185 countries. The study estimated the age-standardized incidence and mortality rates per 100,000 person-years. We projected cases and deaths until 2040 using 2020 incidence and mortality rates as well as global population estimates. In 2020, 905,700 persons worldwide were diagnosed with LC, and 830,200 died as a result. Global LC ASRs ranged between 9.5 and 8.7 per 100,000 individuals, with the greatest rates in Eastern Asia (17.8 new cases, 16.1 deaths), Northern Africa (15.2, 14.5 deaths), and South-Eastern Asia (13.7, 13.2). In 46 and 90 nations, LC was one of the top three or five causes of cancer death. ASR incidence and fatality rates were higher in males than in women globally (male:female ASR ratio: 1.2-3.6). Experts expect a 55.0% increase in LC incidence between 2020 and 2040, reaching 1.4 million in 2040. LC might kill 1.3 million people in 2040, up 56.4% from 2020. The frequency of LC varies across the globe. In 46 and 90 nations, LC was one of the top three or five causes of cancer death. As the world's population grows, we anticipate more cases and deaths in the coming two decades. Prioritizing control measures can help prevent PLC from certain causes, and the expected increase in cases may demand more resources for LC care. LC is a significant cause of death in many nations, and its incidence is expected to rise. This emphasizes the need to implement preventive measures throughout all communities [20].

The incidence of LC is rising in Western countries, most likely due to rising rates of obesity, diabetes, and physical inactivity. According to research, exercise can reduce the incidence of HCC by up to 45% [16]. Obesity [22], diabetes [23], and physical inactivity [24] have been common over the last decade.

Biliary carcinoma is rare in Saudi Arabia; hence, there is minimal literature on it. The prognosis for gallbladder adenocarcinoma, as well as intrahepatic and extrahepatic CCA, is dismal. There is a scarcity of data on BTC in Saudi Arabia. A study of 155 patients discovered 75 with intrahepatic CCA and 74 with gallbladder adenocarcinoma. Females accounted for 63% of patients. Moreover, 59% of the participants in the study were overweight or obese. The majority of patients (62%) had Eastern Cooperative Oncology Group performance in Category 2 or lower. A genetic profile was available in 20% of CCA cases. The CA19-9 tumor marker was high in 57% of patients (>37 µmol/L). De novo metastasis affected 82% of patients. One-third of patients (33%) were unfit for first-line chemotherapy and received the best possible supportive treatment. 53% of fit patients received platinum and gemcitabine, 38% gemcitabine alone, and 9% 5-fluorouracil. Few patients lived 12 months or longer (16%). There is a substantial correlation between neutrophil-to-lymphocyte ratio (NLR) and progression-free survival (PFS). The median PFS for the NLR >3 group was 3.5 months (95% CI: 1.2-6 months), while for the NLR <3 group, it was 9.8 months (95% CI: 7.5-12 months, p = 0.019) [25].

Common risk factors in Saudi Arabia

Limited studies directly establish the relationship between hepatobiliary cancers and associated risk factors in Saudi Arabia. However, some research indicates that the most prevalent risk factors for LCs include HCV (32%), NAFLD at 30%, and HBV at 26% [13].

Viral Hepatitis

Chronic viral hepatitis (HBV and HCV) is still the leading cause of LC worldwide. Viral-related HCC leads to liver inflammation, oxidative stress, and disruption of the cell signaling system. HBV is more carcinogenic than HCV because it integrates into cell DNA and can withstand nucleotide analog suppression. Patients with cirrhosis and “high-risk” chronic HBV infection should get six-month ultrasounds. Antiviral therapy lowers the risk of HCC; however, even after virological suppression or cure, individuals with advanced chronic liver disease should be monitored. Patients with chronic HBV can use several scores to stratify their risk of developing HCC. We need markers/scores to predict long-term HCC risk in HCV-related liver disease patients who respond effectively to direct-acting antivirals [26].

In a Saudi study, male participants had a greater prevalence of hepatitis virus infection (HBV 1.9%; HCV 0.4%) than female participants (HBV: 1.43%; HCV: 0.2%) [27]. In 2014, scientists estimated that 101,000 Saudis had HCV, although only 20% were diagnosed. The base scenario for 2030 anticipates 103,000 viremic cases, 470 HCC cases, 1,300 decompensated cirrhosis cases, 15,400 compensated cirrhosis cases, and 670 liver-related deaths. By 2030, high-efficacy treatment alone was anticipated to result in 80,700 cases of viremic illness, 350 cases of HCC, 480 liver-related fatalities, 850 cases of decompensated cirrhosis, and 11,500 cases of compensated cirrhosis, respectively. In 2030, an intensive treatment approach will result in 1,700 viremic cases, one HCC case, 20 liver-related deaths, and five and 130 instances of decompensated and compensated cirrhosis, respectively. Delaying this strategy by one year will result in 360 additional fatalities by 2030. HCV prevalence in Saudi Arabia stays stable, whereas severe liver disease and mortality rates increase. Increased treatment efficacy and the number treated would have a greater impact than either alone. The anticipated benefit will improve illness prognosis, resource allocation, and HCV control. A nationwide plan may necessitate additional screening and diagnosis [28].

However, HCV infection in Saudi Arabia ranges from 0.4% to 1.1%, with an average prevalence of 0.3%, increasing the incidence of HCC and its associated morbidity and mortality. Between 1996 and 2006, Saudi Arabia had 437,000 HCV cases, which are anticipated to increase to 103,000 by 2030, resulting in an increase in HCC, decompensated and compensated liver cirrhosis, and liver-related mortality [29-32]. Co-infection with HBV and HCV is related to higher comorbidities, severity of liver disease, and risk of HCC, making it more serious than either virus alone [33].

Cirrhosis

Alcohol misuse, metabolic dysfunction-associated steatotic liver disease (MASLD), and viral hepatitis can all result in liver fibrosis, cirrhosis, and cancer. Hepatic fibrogenesis occurs when several types of resident and nonresident liver cells interact in intricate ways. This results in the accumulation of ECM and organ failure. Changes in transcription and protein synthesis, similar to the Warburg effect in cancer cells, alter cell phenotypes and functions. This is because substrate metabolism, including glucose and lipid metabolism, must adjust. Nuclear receptor signaling, autophagy, ferroptosis, the unfolded protein response, and endoplasmic reticulum stress all regulate cellular activity and metabolism. These metabolic alterations are required for macrophage, lymphoid, and hepatic stellate cell inflammatory and fibrogenic activity. Modulation of these pathways enables novel liver fibrosis treatments to halt or reverse development [34]. NAFLD and NASH will be the leading causes of liver disease in Saudi Arabia as obesity and type 2 diabetes mellitus (T2DM) become more prevalent. Advanced liver disease and NAFLD/NASH mortality rates in Saudi Arabia are expected to climb due to high adult obesity and diabetes rates, as well as an aging population. Through 2030, the prevalence of NAFLD will increase alongside obesity and diabetes. Saudi Arabia predicts 12,534,000 NAFLD cases by 2030. NASH cases grew more than NAFLD cases as the population aged and the condition progressed. In Saudi Arabia, compensated cirrhosis and advanced liver disease patients are anticipated to triple by 2030, with 4,800 incident liver deaths [35].

NAFLD

NAFLD is the most prevalent chronic liver disease worldwide. The proportion of NAFLD patients who develop cirrhosis and require risk assessment and HCC surveillance is responsible for the majority of the healthcare burden. NAFLD is anticipated to become the leading cause of cirrhosis and HCC worldwide; nevertheless, noncirrhotic HCC makes surveillance difficult. In NAFLD, extrahepatic cancer fatalities outnumber HCC deaths. Unlike HCC, NAFLD increases the risk of extrahepatic cancer regardless of the state of liver fibrosis. Extrahepatic carcinoma could be a significant health and economic issue, given NAFLD affects around 30% of individuals worldwide [36,37]. From 2012 to 2019, the prevalence of MASLD in Saudi Arabian children and adults increased from 28.02% (n = 8.34 million) to 33.11% (n = 11.83 million), with an APC of +2.43% (95% CI: 2.33-2.54%) [38].

Diabetes

T2DM is a significant risk factor for HCC. ECM mechanical alterations are linked to cancer development. In cirrhosis, stiffness encourages HCC development. Advanced glycation end-products (AGEs) develop in the ECM in T2DM, although their impact on HCC in noncirrhotic cases is uncertain. In human and animal models, AGEs enhance collagen architecture and ECM viscoelasticity, resulting in faster stress relaxation and viscous dissipation but not stiffness. High AGEs, viscoelasticity, and oncogenic β-catenin signaling promote HCC induction. AGE formation can be inhibited, AGER1 can be restored, or AGE-induced collagen crosslinks can be broken. All of these things reduce viscoelasticity and HCC growth. Matrix study and computational modeling reveal that an AGE-bundled collagen matrix with shorter fiber lengths and greater heterogeneity diminishes interconnectivity while increasing viscoelasticity. Higher viscoelasticity increases the spread and invasion of HCC cells via the integrin-β1-tensin-1-YAP pathway, as demonstrated in animal and 3D cell cultures. These findings demonstrate that changes in structure produced by AGEs make the ECM more flexible and accelerate cancer progression in living beings, regardless of how rigid the ECM is [39].

A cross-sectional survey of 5000 Saudis from 30 primary healthcare facilities in the Ḥaʼil Region was carried out. The overall diabetes prevalence in Ḥaʼil was 31.1%. Male prevalence was 32.6%, whereas female prevalence was 29.6% (P < 0.0001). Diabetes risk increases with age and BMI (p < 0.0001) [23].

Hemochromatosis

Patients with hemochromatosis (HFE) have an increased chance of developing HCC. HFE-HCC biology is unknown, and evidence on whether such patients have a worse prognosis is inconsistent. The aggressive clinical history of HFE-HCC patients gives the first indication that their tumors contain more progenitor markers [40].

Aflatoxins

Despite the existence of food safety regulations in numerous countries, significant levels of aflatoxin B1 (AFB1)-DNA adducts are present in both normal and tumorous tissues of cancer patients. Cytochrome P450 enzymes convert AFB1 into AFB1-8,9-epoxide, which subsequently binds to DNA. This metabolite spontaneously and irreversibly forms highly mutagenic DNA adducts on guanine residues. The mutation of ATM kinase induced by AFB1 results in a reduction of G2/M cell cycle checkpoint activation. A-T mutations impair the repair of DNA double-strand breaks, resulting in genomic instability and heightened cancer risk. The primary point mutation in AFB1 is a G-to-T transversion, linked to a mutation in the p53 gene. The prevalence of TP53 mutant DNA in cases of AFB1-associated HCC suggests a correlation between AFB1 exposure and increased risk [41-43]. Food commodities in Saudi Arabia are vulnerable to AF contamination due to inadequate storage practices and the prevalent warm, humid climate in many areas. A study conducted in Saudi Arabia, which analyzed 2,388 food samples, found that 12.1% were contaminated with aflatoxins, with the highest concentration of total aflatoxins identified in the nut and seed category [44].

Smoking and Alcohol Consumption

Cirrhosis, HCC, cholelithiasis, and pancreatitis are serious global health issues. Researchers routinely investigate the health consequences of lifestyle choices such as smoking, alcohol drinking, and coffee consumption. Confounding factors and reverse causality are common obstacles in observational studies, making it difficult to demonstrate causal correlations. A genetic predisposition to tobacco smoking is associated with increased risks of acute pancreatitis, alcoholic hepatitis, chronic pancreatitis, cirrhosis, gallstones, LC, and pancreatic cancer. Alcohol use has been linked to acute pancreatitis, chronic pancreatitis, alcoholic liver disease, hepatic malignancy, and cholangitis. Coffee consumption showed minimal associations and had a minor protective effect against NASH [45]. A Saudi study found that the prevalence of tobacco use and alcohol use was 30.3% and 7.5%, respectively [46,47].

Body Weight

Several observational studies have linked obesity to PLC, although the causality and risk implications of certain obesity markers are unclear. We investigated the risk of PLC using a two-sample Mendelian randomization analysis of genetically determined liver fat, visceral adipose tissue (VAT), and BMI. We obtained exposure summary statistics from two genome-wide association studies (GWASs) involving the UK Biobank imaging cohort and the Genetic Epidemiology Research on Adult Health and Aging cohort. We calculated GWAS summary statistics for PLC using the FinnGen consortium R7 release data, which contained 304 cases and 218,488 controls. Our primary analysis was inverse-variance weighted, and sensitivity testing verified the strength of our findings. The study found that genetically determined liver fat and VAT, not BMI, increased the risk of PLC. This shows that visceral fat distribution is a more accurate predictor of clinical risk than in vitro measures [48]. Between 1990 and 2019, the prevalence of overweight and obesity among Saudi men and women increased. Adult prevalence topped 60%, while children and adolescents had rates ranging from 20% to 60%, indicating a continued trend [49]. A study examined the prevalence of obesity, physical activity, and dietary habits among Saudi adults in the Makkah region of Saudi Arabia, finding that 32.8% of the population was overweight and 23% obese. Obesity and overweight are strongly linked to sociodemographic variables, necessitating focused intervention measures to address the obesity epidemic [50].

Family History

Environmental variables have a substantial impact on health outcomes; nevertheless, people with a family history of LC are at a higher risk of developing HCC. Large cohorts of people of European ancestry have not been routinely studied utilizing genome-wide techniques. The GWAS identifies new genetic risk factors for nonviral HCC in European Americans residing in North America. This indicates that the condition is highly inherited. Genetic factors in HCV-positive HCC have been found. These findings emphasize the role of genetic vulnerability in the development of HCC [51]. A study in Saudi Arabia used whole genome sequencing to investigate the genetic features of patients with HCC. The findings suggest that the majority of HCC patients have cancer-related genetic variations, and the altered pathways observed in these individuals share significant commonalities [52].

Anabolic Steroids

Anabolic steroids can cause hepatocellular adenomas. Hepatocellular adenomas typically lead to HCC [53]. The recreational use of anabolic-androgenic steroids (AAS) is becoming a major global public health concern. Research on health awareness and understanding is limited in underdeveloped nations such as Saudi Arabia. These included 29.3% who had used AAS and 53.5% who had heard about it, most likely from friends. Most study respondents (53.2%, 51.1%, and 45.5%) understood how AAS impacts muscle mass, body weight, and strength. In contrast, a larger proportion of study participants were unaware of the negative effects of AAS. AAS was offered to 43.2% of research participants, and 68.7% believed they were readily available. Moreover, 90.1% of gym users had never used narcotics or psychotropic medications. A regression study found a significant link between the use of AAS and “weightlifting practice,” with the following ORs: OR (95% CI) = 1.9 (1.02-3.61), P = 0.044; OR (95% CI) = 7.8 (4.05-15.03), P < 0.0001; OR (95% CI) = 7.5 (3.78-14.10), P < 0.0001. The study suggests that gym users are encouraged to use anabolic-androgenic drugs [54].

Management

HCC, the world’s sixth most common cancer, is associated with high morbidity and mortality. Intermediate- and advanced-stage HCC pose a significant healthcare burden in Saudi Arabia, yet little is known about its unmet needs and treatment options. This article fills gaps and provides professional consensus on Saudi Arabian unresectable HCC management strategies. HCC accounted for the majority of LC cases in Saudi Arabia, owing to increased chronic viral infections and lifestyle risks. Most Saudi doctors utilize the Barcelona Clinic Liver Cancer Criteria to detect and stage HCC. The majority of Saudi HCC patients are diagnosed at middle or advanced stages, which generally leads to a poor prognosis and restricted therapeutic options. To address HCC in Saudi Arabia, evidence-based surveillance, multidisciplinary diagnosis, and more treatment options are required [55].

Because of the high prevalence of HCC in Saudi Arabia, as well as the challenges of early and accurate diagnosis, evidence-based management, and appropriate referral, the Saudi Association for the Study of Liver Diseases and Transplantation established a multidisciplinary task force to evaluate and update the Saudi Gastroenterology Association’s guidelines. The Saudi Oncology Society reviewed, adopted, and approved these guidelines as its official HCC guidelines. The Saudi HCC guidelines revision committee included hepatologists, oncologists, liver surgeons, transplant surgeons, and interventional radiologists. Two task force members edited the guidelines. All published HCC epidemiology, natural history, risk factors, diagnostic, and management studies were reviewed. All literature was critically assessed, and evidence was ranked according to its strength. Members thoroughly studied the materials and ideas before reaching an agreement. All of the recommendations in these guidelines were based on the greatest research, but they were customized to the needs of Saudi patients. We expect that these guidelines [55] will improve the multidisciplinary treatment needed for HCC patients and their overall quality of care. Evidence-based surveillance approaches, a multidisciplinary diagnosis approach, and increased access to treatment choices are critical for managing HCC in Saudi Arabia. This includes extensive future studies into all aspects connected with hepatobiliary carcinoma in this country.

## Conclusions

Hepatobiliary cancers, particularly LC, are prevalent in Saudi Arabia. The main risk factors for hepatobiliary cancers in Saudi Arabia include viral hepatitis (primarily HCV, then HBV), NAFLD, T2DM, obesity, and tobacco use, among others. The timely deployment of hepatobiliary preventative strategies and early detection strategies are deemed critical. These include early-stage patient diagnosis and the management of major risk factors. The paucity of gallbladder and bile duct cancers makes it difficult to determine their impact in Saudi Arabia, necessitating further research.
